# Giant cell tumor - distal end radius: Do we know the answer?

**DOI:** 10.4103/0019-5413.32046

**Published:** 2007

**Authors:** Yogesh Panchwagh, Ajay Puri, Manish Agarwal, Chetan Anchan, Mandip Shah

**Affiliations:** Bone and Soft Tissue Unit, Dept. of Surgical Oncology and Tata Memorial Hospital, Parel, Mumbai, India; Orthopaedic Oncologist, Dept. of Surgical Oncology, Tata Memorial Hospital, Parel, Mumbai, India

**Keywords:** Campanacci grade, distal end radius, function, giant cell tumor, recurrence

## Abstract

**Background:**

The distal end of the radius is one of the common sites of involvement in giant cell tumors (GCTs) with reportedly increased propensity of recurrence. The objective of the present analysis was to study the modalities of management of the different types of distal end radius GCTs so as to minimize the recurrence rates and retain adequate function.

**Materials and methods:**

Twenty-four patients of distal end radius GCTs treated between January 2000 and December 2004 were retrospectively reviewed. Nineteen cases were available for follow-up with an average follow-up of 37.5 months. There was one Campanacci Grade 1 lesion, nine Grade 2 and 14 Grade 3 lesions. Thirteen (54%) of these patients were treated elsewhere earlier and presented with recurrence. The operative procedures that were performed were: curettage and cementing (five), curettage and bone grafting (seven), excision and proximal fibular arthroplasty (two), excision and wrist arthrodesis (nine) and excision of soft tissue recurrence (one).

**Results:**

Functional status was evaluated using Musculo Skeletal Tumor Society scoring system which averaged 78%. The recurrence rate was 32%. Complications included local recurrence (six), nonunion at the graft bone junction (one), infection (one), deformity (two), stiffness (two), subluxation (two) and bony metastasis (one).

**Conclusions:**

The majority of patients undergoing curettage were either Campanacci Grade 1 or 2. Patients undergoing curettage and reconstruction had a better functional result (82%) as compared to arthrodesis or fibular arthroplasty (69%). Previous intervention did not appear to increase the recurrence rates. Even though complications occur, judicious decision-making and an appropriate treatment plan can ensure a satisfactory outcome in the majority of cases.

Giant cell tumor (GCT) is a benign, locally aggressive neoplasm of bone which is composed of sheets of neoplastic, ovoid, mononuclear cells interspersed with uniformly distributed large osteoclast-like giant cells.^1^

GCTs comprise about 4-5% of all primary bone tumors and about 20% of benign bone lesions. The peak incidence is between 20 to 45 years of age.^1^ Seventy per cent of the cases of GCT fall in this age group.^2^ It is rarely found in the less than 10 age group. GCT commonly affects the ends of long bones. The distal femur, proximal tibia, distal radius and proximal humerus are the commonly affected sites in the appendicular skeleton, while the sacrum is the commonly affected site in the axial skeleton.^1^ Rarely, GCTs are multicentric or found primarily as lesions arising in the soft tissues.

The lower end of the radius is the third common site to be affected. The distal radius plays a significant role in the radio-carpal articulation and hence in the function of the hand. It is always a challenge to reconstruct the defect caused by excision of the distal radius tumors. The complex anatomy and the need to obtain acceptable functional outcome with good disease clearance creates a dilemma in the treatment of the GCTs of the lower end of the radius. Various treatment modalities are advocated in the literature. These include:

1. Extended curettage,^3^ with or without reconstruction using autogenic/allogenic bone grafts or polymethyl-methacrylate^4^,^5^
2. Resection and reconstruction with vascularized or nonvascularized proximal fibula (fibular head arthroplasty)^6^–^16^
3. Resection with partial wrist arthrodesis (radio-scapho-lunate arthrodesis) using a strut bone graft^17^
4. Resection and complete wrist arthrodesis using an intervening strut bone graft.^18^–^21^

Thorough curettage and complete excision is the single most important factor to prevent recurrence. There are few studies in the literature which have tried to analyze the treatment modalities with respect to the radiological grade of the distal radius GCT.^3^,^5^,^6^,^22^–^24^

Campanacci Grade 1 and 2 lesions usually do well with extended curettage alone or with bone graft or cement reconstruction. They also are found to have the best functional results. Campanacci Grade 3 lesions require resection of the entire lesion and reconstruction when the extraosseous soft tissue component is large.

We undertook a retrospective study of the surgically treated giant cell tumors of the distal radius to analyze the treatment patterns, the recurrence rates, the complications and the functional outcome.

## MATERIALS AND METHODS

A retrospective analysis of 24 cases of GCT of the distal end of the radius, treated from January 2000 to December 2004 was done. These included the cases treated primarily as well as the cases with a recurrence after undergoing surgery outside. All the lesions were biopsy-proven GCTs. The minimum follow-up was of 18 months after the surgery. Radiological grading of the lesions was done as per Campanacci grading.^25^ The radiographs and the computed tomography (CT) scans and magnetic resonance imaging (MRI) scans when available were studied.

Grade 1 Campanacci lesion has well-marginated border of a thin rim of mature bone with the cortex being intact or slightly thinned, but not deformed. Grade 2 lesion has relatively well-defined margins but no radio-opaque rim, with the rim being thin and moderately expanded but still intact. Grade 3 tumor was one with fuzzy borders, with extension into soft tissues which did not follow the contour of the bone and was not limited by an apparent shell of reactive bone [Figure 1a, b, c].

**Figure 1A:**
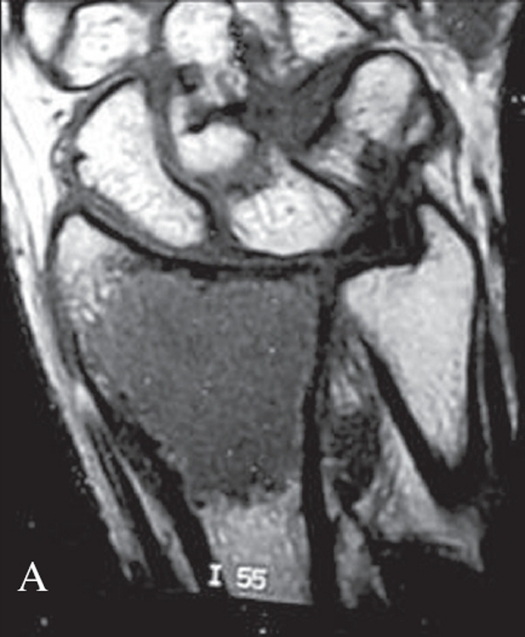
Campanacci grade 1 lesion: Bone contour maintained, purely intra-osseous lesion. Thinning of cortex may be seen

**Figure 1B:**
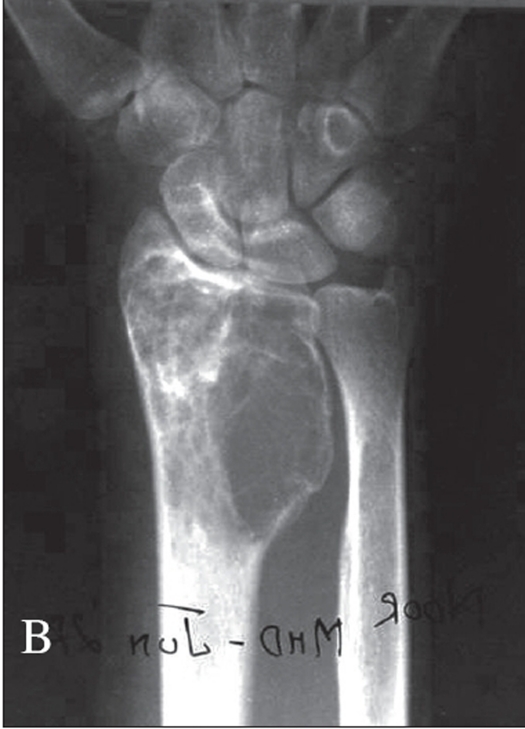
X-ray (antero-posterior) of wrist showing Campanacci grade 2 lesion: Cortical expansion with thin bony rim; but no breach

**Figure 1C:**
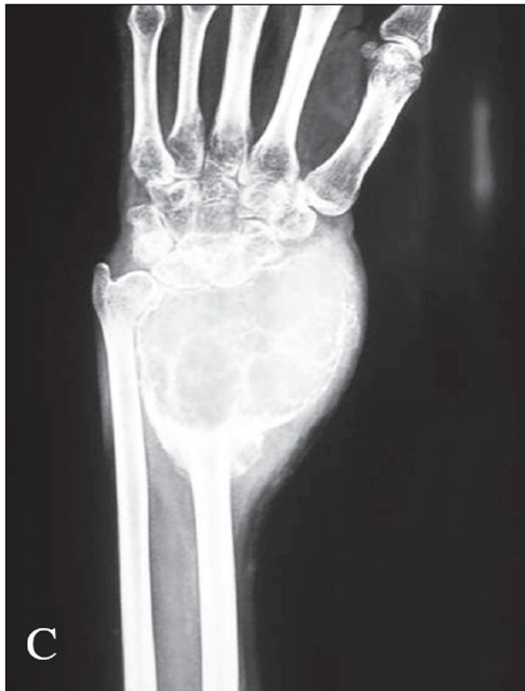
X-ray (antero-posterior) of wrist showing Campanacci grade 3 lesion: Deformed contour, cortical breach with soft tissue extension

The types of surgeries undertaken were individualized, with the type of surgery based on the clinico-radiological and intraoperative findings. Resection was required when it was felt that bone salvageability by intralesional methods would result in such severe mechanical compromise that skeletal integrity was unlikely to be maintained or unlikely to be restored after healing. Some Campanacci Grade 3 lesions in spite of soft tissue extension had adequate bone stock for residual skeletal stability even after extended curettage.

**Figure 2:**
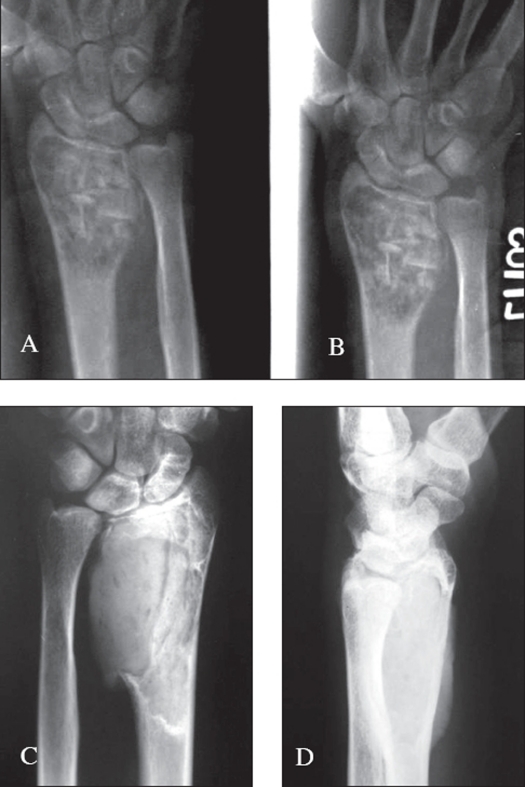
A) Case 1: Grade 2 lesion - Distal radius G.C.T, Treated elsewhere with curettage and bone grafting. B) Had a recurrence, Repeat Curettage and reconstruction done with bone cement. C) Follow up A.P. x-ray at a four year. D) Lateral x-ray of the same patient at four year follow up showing no recurrence of lesion and good subchondral bone

In the cases treated with curettage, a thorough curettage was ensured by making adequate-sized cortical windows and using sharp curettes. The surrounding tissues were protected using hydrogen peroxide-soaked gauze pieces to avoid contamination due to spillage. A burr was used to break any bony ridges wherever necessary. Following this, the cavity walls, wherever suitable, were treated with phenol taken on cotton swab-sticks to ensure microscopic disease clearance. Absolute alcohol was then used to dissolve the phenol. The reconstruction of the defect was then done using either bone cement or autogenous bone grafts [Figure 2].

For the cases which needed resection an en bloc resection was done. After the en bloc resection, the wrist was arthrodesed using a strut graft from the iliac crest or fibula or ulna. In a few cases the arthrodesis was done by centralizing the carpus over the ulna. Suitable implants were used for the arthrodesis [Figures 3 and 4].

**Figure 3A:**
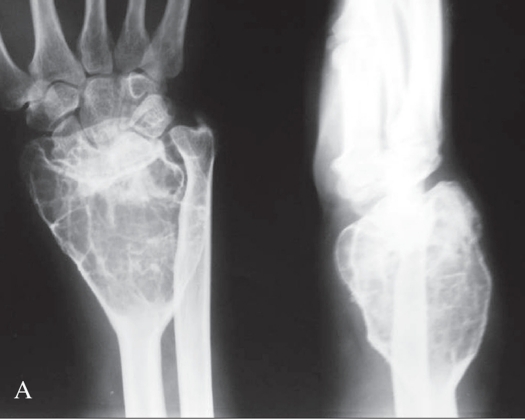
X-ray (A.P. and lateral) of distal radius shows campanacci grade 2 lesion

**Figure 3B:**
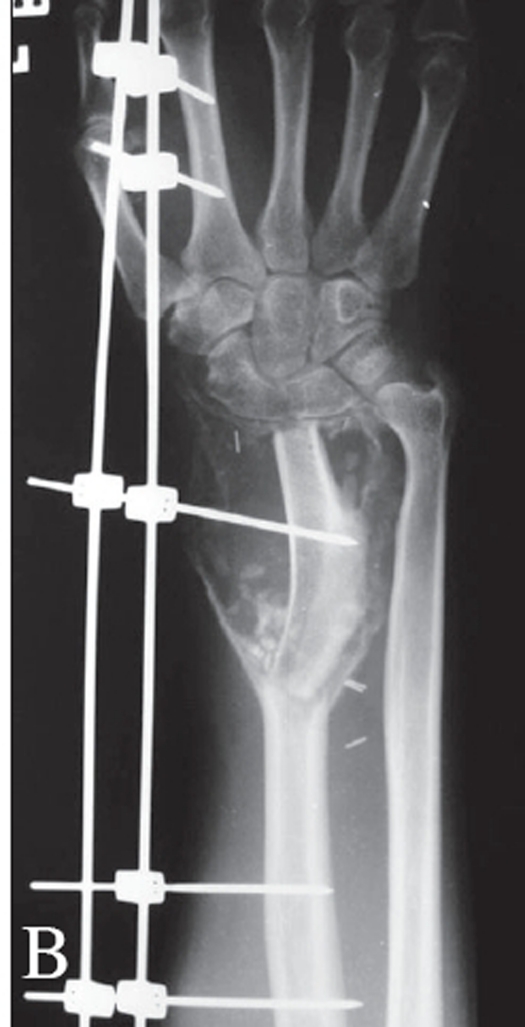
X-ray of the same patient treated with extended curettage and reconstruction with iliac crest strut bone graft along with morsellised grafts. Stabilization was achieved by an external fixator

**Figure 3C, D:**
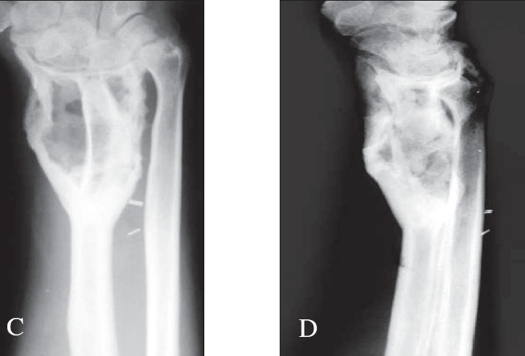
X-rays (A.P. and lateral) of the same patient showing good consolidation

**Figure 4:**
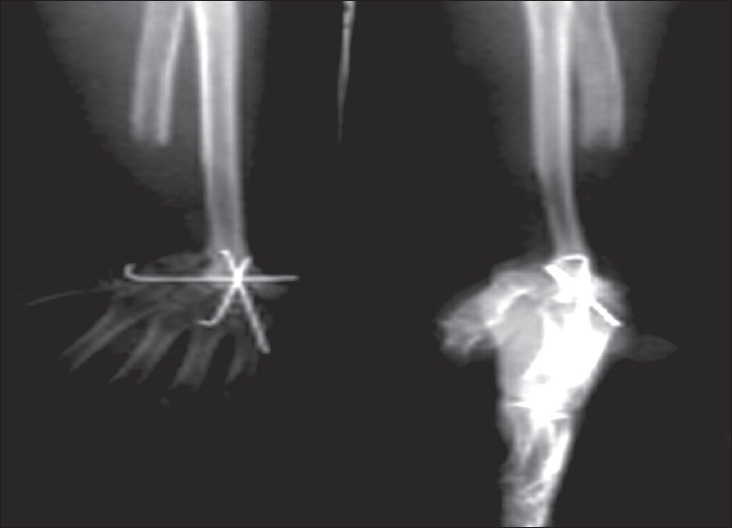
A.P. & Lateral x-ray at a two year follow up of a grade 3 lesion treated with excision and wrist arthrodesis by centralization of the carpus on the ulna. Good fusion seen even with K wire stabilization with advantage of retained prono-supination

On follow-up, the patients were examined clinically and radiologically for any sign of local recurrence and impending complication viz. gap nonunion. The associated recurrence rates, disease-free intervals and the related complications were studied. The functional scoring of the outcome was done using the Musculo Skeletal Tumor Society system. This scoring system measures the function in the upper extremity by assigning points (0-5) under six different headings. These headings are pain, function (in view of restriction of activities), emotional acceptance, hand positioning, manual dexterity and ability of lifting weight. The functional score is expressed in percentage of the actual points scored out of the total 30.

## RESULTS

A total of 263 GCTs were treated between January 2000 and December 2004. The distal end of the radius was affected in 26 (10%). Out of these 26 cases, two were pure soft tissue recurrences without any bony involvement and hence excluded from the analysis. Of the 24 cases analyzed, 13 were male and 11 were female patients. The side affected was right in 10 and left in 14. The age distribution ranged from 15 years (youngest) to 66 years (oldest) with a mean of 36 and median of 32 years. The commonest presenting symptom was swelling (n=13), followed by swelling and pain (n=10). Pain was the only presenting complaint in one case. Fourteen patients had been treated earlier elsewhere and had presented to us with a recurrent lesion; whereas ten had presented primarily. In four of the 14 patients treated elsewhere earlier, two or more surgeries had been performed for the distal radius GCT.

The lesions were graded radiologically as per the Campanacci grading system. Only one of the lesions was Grade 1; while nine were Grade 2 and 14 were Grade 3. The surgeries performed were: curettage and bone grafting (n=7), curettage and cementing (n=5), enbloc resection and fibular arthroplasty (n=2), enbloc resection and wrist arthrodesis (n=9) and excision of soft tissue recurrences (n=1). Fibular or iliac crest graft was used for the arthrodesis in four patients while ulna was transposed in five cases. No allografts were used. The distribution of the type of surgeries performed for the prior treated and untreated patients is shown in the Table 1.

**Table 1:** Surgery performed for distal radius giant cell tumor

Type of surgery	Total no.	In untreated cases	In prior treated cases
Resection + fibular arthroplasty	2	1	1
Curettage + cementing	5	3	2
Curettage + bone grafting	7	4	3
Excision of soft tissue recurrance	1	0	1
Resection + wrist arthrodesis	9	3	6
Fibular./iliac crest	4	2	2
Ulnar transposition	5	1	4

The Grade 1 and 2 lesions (n=9) were treated with curettage and reconstruction with either cementing (n=4) or bone grafting (n=5). One case with Campanacci Grade 2 who had very poor bone stock and extensive cortical thinning was treated with enbloc resection. The Grade 3 lesions were treated with enbloc excision and reconstructed either with proximal fibular graft (n=2) or with wrist arthrodesis (n=8). One patient having soft tissue recurrence was treated with marginal excision of the recurrent lesion [Table 2]. Three cases with adequate bone stock were selected for curettage. Three patients with Campanacci Grade 3 lesions in whom the soft tissue extension was minimal, had adequate bone stock which enabled treatment with extended curettage.

**Table 2:** Surgery performed as per the radiological grading

Grade (no. of cases)	En bloc excision + prox. fibular arthropalsty	Currettage + bone grafting	Curettage + cementing	En bloc excision + arthrodesis	Excision of soft tissue recurrence
Grade 1 (1)	0	1	0	0	0
Grade 2 (9)	0	4	4	1	0
Grade 3 (14)	2	2	1	8	1

The patients were followed up clinico-radiologically with the follow-up period ranging from 18 months to 71 months, with an average of 37.5 months. Five patients (three from the previously treated group and two from previously untreated group) were lost to follow-up. Amongst the 19 patients who were followed up, six had recurrences. The recurrences occurred at an average of 17 months after surgery. The recurrence rate for the previously treated group was 36% (4/11) whereas that for the previously untreated group was 25% (2/8). The average recurrence rate for the 19 cases of distal end radius GCT was 32% (6/19). Analysis of the recurrences with respect to the radiological grading revealed that there were no recurrences in the Grade 1 group. Two patients (29%) amongst the Grade 2 group (n = 7) had a local recurrence whereas four of (36%) Grade 3 lesions (n = 11)) had recurrence [Table 3].

**Table 3:** Radiological grade and recurrence

No. of cases	Grade I (1)	Grade II (9)	Grade III (14)
Available for follow-up	1	7	11
Rec. in fresh cases	0	1 of 4 (25)	1 of 3 (33)
Rec. in recurrent cases	-	1 of 3 (33)	3 of 8 (38)
Total recurrences	0	2 (29)	4 (36)

Recurrence: 6/19 = 32%, Figures in parentheses are in percentage, Rec.= Recurrence

The functional scoring of the outcome for the 19 patients, who followed up, was done at the last follow-up of each patient (Range: 18 months to 71 months; Average: 37.5 months) using the Musculo Skeletal Tumor Society System. The functional score in this study ranged between 60-93% with the average being 78%. The patients treated with curettage and reconstruction and those treated for soft tissue excision had the best functional outcome with scores of around 82%. The patients who had undergone en bloc resection with wrist arthrodesis fared well with scores around 74%. The lesions treated with enbloc resection and proximal fibular replacement had the least functional scores which ranged around 69% [Table 4].

**Table 4:** Type of surgery and functional score

Type of surgery	No. of cases	Functional score %
En bloc resection + fibular arthroplasty	2	69
Curettage + cementing	5	83
Curettage + bone grafting	7	82
Excision of soft tissue recurrence	1	80
Enbloc resection + wrist arthrodesis	9	74%

**Figure 5:**
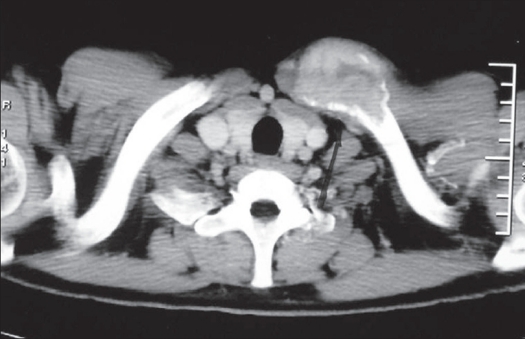
Isolated Skeletal metastasis at the medial end of left clavicle in a treated distal radius GCT seen 4 years after the surgery

Complications as a result of the disease or the treatment modality did occur. There were six recurrences as outlined earlier. Nonunion at the graft-host bone junction was observed in one case while one had a delayed union at the host bone-graft junction. The delayed union was seen in a case where a nonvascularized fibula was used for arthrodesis. The nonunion was in a case where nonvascularized ulnar strut was used for radio-carpal arthrodesis. This was a re-surgery for local recurrence. Deformity was noted on follow-up in two cases. Stiffness of the fingers and salvaged wrist joint was seen in one case each. The other complications included superficial infection at iliac crest (n=1), carpal subluxation (n=1), posttraumatic fracture-dislocation of the radio (fibulo)-carpal joint (n=1), bony metastasis to ipsilateral clavicle (n=1) [Figure 5] and implant-related pain (n=1).

Ten patients had to undergo second surgery. These included the six patients with recurrences. The patient with junctional nonunion required autogenous bone grafting. The patient who presented with an ipsilateral clavicular metastasis six years after the first surgery was treated with clavicular excision. One patient who had undergone proximal fibular arthroplasty developed a posttraumatic fracture dislocation. He had to be treated with ulno-carpal arthrodesis. In the patient with implant-related pain, a prominent K wire had to be removed.

## DISCUSSION

There are various anatomical restrictions in treating a distal radius giant cell lesion. It is closely associated with the radio-carpal and the radio-ulnar joints. The muscle cover is relatively limited. There are various important vessels, nerves and tendons that need to be protected so as to preserve optimum hand function. All these factors need to be taken into consideration while treating distal radius GCT so as to strike the right balance between complete disease clearance and retaining good hand function. At the same time, the recurrence and the complication rates need to be kept low.

Most authors agree that the completeness of the curettage and excision is the single most important factor to prevent recurrence.^3^,^4^,^24^ A Campanacci Grade 1 or 2 GCT of the distal radius is usually treated by curettage and reconstruction with either polymethyl methacrylate (PMMA) or bone grafts. The recurrence is easier to be noticed with a cement reconstruct and it also gives immediate structural stability. The exothermic reaction that occurs while the cement cures is also supposed to increase the tumoricidal effect.^24^ However, there are recent reports throwing light on the cartilaginous degeneration arising thereof. Bone grafts are supposed to be more biological though an early recurrence is difficult to be noticed with the same. Blackley *et al*^4^ have stated that despite the high rates of recurrence reported in the literature after treatment of GCT with curettage and bone grafting, the results of their study suggest that the risk of local recurrence after curettage with a high-speed burr and reconstruction with autogenous +/− allograft bone is similar to that observed after use of cement and other adjuvant treatment. Cheng *et al*.^5^ treated Grade III lesions with curettage when the tumor did not invade the wrist, destroy more than 50% of the cortex or break through the cortex with an extraosseous mass in more than one plane. Khan *et al*^3^ have shown that curettage alone is adequate treatment for the majority of patients with GCTs of the distal radius; but some form of stabilization may be required in the presence of extensive bone destruction.

Harness and Mankin^23^ have put forth data which supports the concept that marginal resection and complete distal radial allograft implantation should be used for patients with tumors that have destroyed much of the bone and have extensive soft tissue components (Campanacci Grade 3). They state that curettage and PMMA insertion should be reserved for patients where the structural alteration of the bone is minimal (Campanacci Grade 1, 2).

There are a lot of studies advocating proximal fibular replacement as the reconstructive option after distal radius excision. It has been found to have good functional result in the other series in the literature. 6,16 However, the two cases treated in a similar way in our series didn't duplicate the same results. The functional scores of these two patients were found to be the lowest (69%). One of the patients later underwent arthrodesis due to posttraumatic fracture-dislocation of the carpus. The other patient had wrist stiffness, limited prono-supination and subluxation of carpus.

In our series the recurrence rates were also found to be associated with the grade of the lesion. None of the Grade 1 lesions recurred, whereas the percentage of recurrence was 29% and 36% with Grade 2 and 3 respectively. A prior surgical intervention elsewhere did not affect future recurrence rates significantly. Campanacci Grade 1 and 2 lesions, which were mostly treated with curettage and reconstruction, had the best functional outcome.

We did not find any pulmonary metastases in our patients till the latest follow-up. However, there was one patient with a skeletal metastasis to the ipsilateral clavicle which was excised and the patient currently remains disease-free.

Complication was noticeable in the fibular arthroplasty group in the form of subluxation and stiffness. However, in our study the number of cases with proximal fibular replacement was only two. We had one case of gap nonunion in the patients treated with arthrodesis. Amongst the arthrodesed patients, one had manus valgus deformity, one had finger stiffness and one had superficial infection at the iliac crest site which healed without surgical intervention. Out of the patients treated with curettage and bone grafting, one had collapse and angulation and one patient had to undergo a K wire removal for implant-related pain. The patient with collapse and angular deformity was advised surgery but the patient refused. Thus, in spite of good functional result, one has to be prepared for the complications that may occur.

## CONCLUSION

The more localized lesions are best treated with curettage. Those with extensive cortical destruction and large soft tissue component usually need en bloc resection. Some Campanacci Grade 3 lesions may be treated with intralesional excision with appropriate stabilization if the bone stock permits. A careful clinical and radiological assessment of the distal radius GCT and judicious treatment plan is the key for successful outcomes in these lesions.
